# Musculoskeletal Pain in Gymnasts: A Retrospective Analysis on a Cohort of Professional Athletes

**DOI:** 10.3390/ijerph18105460

**Published:** 2021-05-20

**Authors:** Giacomo Farì, Francesco Fischetti, Alessandra Zonno, Francesco Marra, Alessia Maglie, Francesco Paolo Bianchi, Giuseppe Messina, Maurizio Ranieri, Marisa Megna

**Affiliations:** 1 Department of Basic Medical Sciences, Aldo Moro University, Piazza Umberto I, 70121 Bari, Italy; francesco.fischetti@uniba.it (F.F.); alessandrazonno2014@gmail.com (A.Z.); dr.francescomarra@gmail.com (F.M.); maurizio.ranieri@uniba.it (M.R.); marisa.megna@uniba.it (M.M.); 2 Department of Biological and Environmental Science and Technologies (Di.S.Te.B.A.), University of Salento, Piazza Tancredi 7, 73100 Lecce, Italy; alessia.maglie@studenti.unisalento.it; 3 Department of Biomedical Science and Human Oncology, Aldo Moro University, Piazza Umberto I, 70121 Bari, Italy; frapabi@gmail.com; 4 Department of Psychology, University of Palermo, Via delle Scienze, Ed 15, 90128 Palermo, Italy; giuseppe.messina17@unipa.it

**Keywords:** overload training, wrist pain, injury prevention, overuse, sitting position

## Abstract

Gymnastics athletes are exposed to a high risk of injury, but also of developing musculoskeletal pain. These data are still little investigated in the available scientific literature. An online survey was distributed to 79 professional athletes who practiced artistic and rhythmic gymnastics. The survey collected demographic and anthropometric data, information about the sport practice, the training sessions, the prevalence of musculoskeletal pain gymnastics-related, and lifestyle habits. Musculoskeletal pain had a high prevalence, involving 65 of 79 athletes (82.3%). A significant correlation was found between musculoskeletal pain and the duration of sports practice, both for general pain (*p* = 0.041) and for specific districts: right wrist pain (*p* = 0.031), left wrist pain (*p* = 0.028), right shoulder (*p* = 0.039), left hip (*p* = 0.031), right thigh (*p* = 0.031), and left knee (*p* = 0.005). Another statistical association was found between right wrist pain and BMI (*p* = 0.001), and hip pain and BMI (*p* = 0.030). Hours spent in a sitting position were also correlated with the incidence of pain (*p* = 0.045). Wrist pain and right shoulder pain had a statistically significant association with the age of the athletes (right wrist pain: *p* = 0.038; left wrist pain: *p* = 0.004; right shoulder pain: *p* = 0.035). The more the gymnasts practice this sport, the more likely they are to develop musculoskeletal pain. Increased age and a higher BMI, as well as daily prolonged sitting position, seem to be potential risk factors for the onset of musculoskeletal pain. Future studies could plan training strategies aimed at preventing musculoskeletal pain associated with gymnastics, in order to promote its further spread.

## 1. Introduction

Gymnastics is a grueling sport that requires considerable physical and mental effort for a continuous search for harmony between biomechanics and aesthetics efforts. Current International Gymnastic Federation (IGF) disciplines include rhythmic gymnastics and artistic gymnastics, which is further divided in men’s artistic gymnastics (MAG) and women’s artistic gymnastics (WAG) [1].

Gymnastic elements that must be assimilated and acquired by gymnasts necessarily require the development of coordination, joint mobility, postural adaptation, strength, speed, rhythm, agility, and dynamism. In rhythmic gymnastics, a refined quality of motor control, excellent expression skills, and elegance of the technical gesture are quite important [2,3,4,5].

In order to achieve the proper skills required for a correct execution of sports gestures from an early age, high-performance training is required. The athletes usually train for, on average, 25–30 h per week and, in some cases, 40 h per week. This is due to the high technical demands of this sports discipline [6]. Therefore, it is reasonable to hypothesize that rhythmic and artistic gymnastics are sports that put athletes at risk of musculoskeletal disorders such as wrist pain [5], low-back pain [2,7,8,9,10,11], shoulder pain [12,13], postural disorders [14,15], and many injuries [16,17], all mainly caused by overuse and repeating the same gestures several times for every type of training.

In addition, this sports practice, that usually begins at an early age, lasts throughout the growth period, including the phases of rapid growth [18]; consequently, gymnasts are exposed to injuries and to the onset of musculoskeletal pain (MP) [10] related to sports practice.

Gymnastics is affected by a high incidence of sport-related pain and lesions [7]. Since the number of those who practice these sports has increased over the years [19], there is a risk of an increase in the costs of medical care, so it is crucial to design strategies for the prevention of MP and injuries.

Moreover, it is also interesting for gymnasts to try to understand if and how lifestyles, especially in professional athletes, affect the onset of musculoskeletal pain. This is all the more interesting in a historical period such as the present one, in which the restrictions on the usual sporting activity imposed by the COVID-19 pandemic have led to inevitable postural and musculoskeletal dysfunctions [20,21].

The aim of this study is to determine the prevalence of musculoskeletal pain, differentiated by anatomical districts, in a cohort of artistic and rhythmic professional gymnasts and to investigate the main risk factors involved.

## 2. Materials and Methods

### 2.1. Study Design and Participants

The study model is that of an observational retrospective study.

All gymnasts were professional athletes. An online survey was set up using Google Forms. The survey was distributed by email on 12 June 2020 and was requested to be completed and submitted by 27 June 2020.

Informed consent was obtained from all participants involved in this study; in the case of underage athletes, consent forms were filled by parents (or by holder of the responsibility on the minor).

All the procedures were conducted in accordance with the principles set forth in the Helsinki Declaration.

### 2.2. Procedures

The survey consisted of multiple choice and open-ended questions divided in three different sections:

The first section provided information about the study and contained the informed consent; this section also includes demographic and anthropometric data.

The second section concerned the athletes’ practice and the characteristics of the training sessions.

The third section focused on musculoskeletal pain related to specific sports activities. To define this pain, we gave gymnasts the following definition: “Any pain involving muscles, tendons, and joints that occurs in a manner closely related to the specific sports practice, and that recurs in a cyclical way following the usual gymnastic sessions, in the absence of specific traumas that can justify it”. In order to delve into the origin of MP, we collected data about lifestyle habits that could also affect the onset of MP (daily hours spent in a sitting position, for usual daily activities such as working or studying).

### 2.3. Statistical Analysis

Continuous variables are expressed as mean ± standard deviation and range; categorical variables are expressed as proportions, with an indication of the 95% confidence interval (95% CI), where deemed appropriate. The × 100 person-months incidence rate was calculated using the sports activity time (months) as the denominator and the number of events as the numerator; 95% CI was subsequently indicated.

Univariate logistic regression was used to evaluate the association between dichotomic outcomes and determinants; the odds ratio (OR) was calculated with the indication of 95% CI.

A *p*-value < 0.05 was considered significant for all tests.

All the statistical analyses were conducted using the Excel Real Statistics Resource Pack (Microsoft Corporation, Redmond, WA, USA).

## 3. Results

The cohort consisted of 79 athletes: 54 rhythmic gymnasts, 24 WAG athletes, and 1 MAG athlete, whose demographic characteristics are described in Table 1.

As described in Table 2. A total of 65 out of 79 athletes (82.3%) experienced recurrent MP related to gymnastics practice.

The period of sports practice is strongly correlated with the incidence of pain (OR = 1.01; 95% CI = 1.01–1.04; *p* = 0.041). In particular, the athletes who have practiced for many years are more affected by painful wrist syndromes: wrist pain has a statistically significant association with the period of sports practice (right wrist pain: OR = 1.02, 95% CI = 1.01–1.03, *p* = 0.031; left wrist pain: OR = 1.02, 95%, CI = 1.01–1.04, *p* = 0.028), but also with the age of the athletes (right wrist pain: 1.23; 95%, CI = 1.01–1.50, *p* = 0.038; left wrist pain: 1.46, 95% CI = 1.13–1.90, *p* = 0.004). Another statistical association is between right wrist pain and BMI (OR = 1.72, 95% CI = 1.24–2.39, *p* = 0.001).

The same evidence was found for right shoulder pain. Gymnasts more prone to musculoskeletal pain in this anatomical region are the older athletes (OR = 1.30, 95% CI = 1.02–1.66, *p* = 0.035) and the ones who have practiced sports for a longer time (OR = 1.02, 95% CI = 1.01–1.04, *p* = 0.039); at the same time, the hip pain is associated with the period of sports practice (OR = 1.02, 95% CI = 1.01–1.03, *p* = 0.031) and with BMI (OR = 1.47, 95% CI = 1.04–2.07, *p* = 0.030).

There is also a significant association between right thigh pain and the period of sports practice (OR = 1.02, 95% CI = 1.01–1.03, *p* = 0.031), and also between left knee pain and the period of sports practice (OR = 1.02, 95% CI = 1.01–1.04, *p* = 0.005).

A total of 43.6% of the sample spent more than 4 hours in a seated position; it emerged that the athletes who spent more daily hours in a sitting position were more exposed to MP (OR = 1.91, 95% CI = 1.01–3.59, *p* = 0.045).

## 4. Discussion

We found a significant correlation between musculoskeletal pain and the duration of sports practice, both for general pain (OR = 1,01, 95% CI = 1.01–1.04, *p* = 0.041) and for specific districts: right wrist pain (OR = 1.02, 95% CI = 1.01–1.03, *p* = 0.031), left wrist pain (OR = 1.02, 95% CI = 1.01–1.04, *p* = 0.028), right shoulder (OR = 1.02, 95% CI = 1.01–1.04, *p* = 0.039), left hip (OR = 1.02, 95% CI = 1.01–1.03, *p* = 0.031), right thigh (OR = 1.02, 95% CI = 1.01–1.03, *p* = 0.031), and left knee (OR = 1.02, 95% CI = 1.01–1.04, *p* = 0.005). This evidence is in line with the current scientific literature. In fact, MP is more frequent in high frequency and intensity sports [17,18] and in long sports practice [17]. In 2016, Kamada et al. [19] carried out research into the dose-response relationship between sports activity and MP in adolescents and found that each additional 1 h/wk of sports activity was associated with a 3% higher probability of having pain. Moreover, this evidence is found in relation to the duration of sports practice, regardless of age; therefore, it also concerns younger athletes, such as those belonging to our sample [19]. The longer is the time dedicated to the sports practice, the greater is the biomechanical overload on the musculoskeletal system. Many studies stated the correlation between sports overuse and MP [22,23]. In gymnastics, if subjected to excessive stress, such as excessive load, inadequate preparation, and insufficient rest-recovery phase, the musculoskeletal system can undergo various types of overuse injuries and pain that can affect different musculoskeletal anatomical districts [22]. This is mainly detected in artistic and rhythmic gymnastics athletes. These activities particularly overload certain joints, less interested in most other sports, such as wrists, whose pain incidence increases as participation and level of competition increase [2,24,25,26]. Hence, the definition of “gymnast’s wrist” [27]. DiFiori et al. suggest that a threshold of training intensity may be important in the development of wrist pain: they found that gymnasts with wrist pain trained more hours per week and trained at a higher skill level [5].

As the duration of sports practice increases, MP increases in another district of the upper limb as well, such as the right shoulder (probably more affected due to the prevalence of right-handed gymnasts), and in three districts of the lower limb, which are left hip, right thigh, and right knee. These findings are confirmed in the updated scientific literature [19,27,28].

Our statistical analysis points out that hours spent in a sitting position seem to be correlated with the incidence of pain (OR = 1.91, 95% CI = 1.01–3.59, *p* = 0.045) as well. These are the most important data relating to lifestyles that seem to affect the appearance of pain net of the causes directly attributable to sporting activity. Prolonged sitting is typical among the habits of contemporary society [29]. Referring to our specific sample, made up predominantly of young adolescents, the circumstances related to prolonged sitting are likely the hours spent at school or studying, the time spent at home, for example watching TV, or using a computer and a mobile phone. Often, the sitting position is incorrect, and this could have important implications for the onset of MP; in fact, pain due to prolonged incorrect postures is quite frequent in many districts of the musculoskeletal system, such as the cervical spine, with referred pain to the head, upper limbs [29], and lumbar spine [29,30,31], so education in appropriate sitting postures should be promoted from a young age [29]. It is desirable that future epidemiological studies on larger samples can investigate to what extent this lifestyle factor influences the onset of musculoskeletal pain in those who practice gymnastics in a professional manner, and especially if—and to what extent—sports practice can mutually influence this lifestyle factor.

Wrist pain has a statistically significant association with the age of the athletes (right wrist pain: OR = 1,23, 95% CI = 1.01–1.50, *p* = 0.038; left wrist pain: OR = 1.46; 95% CI = 1.13–1.90, *p* = 0.004). The same evidence was also found for right shoulder pain: gymnasts more prone to musculoskeletal pain in this anatomical region are the older ones (OR = 1.30; 95% CI = 1.02–1.66, *p* = 0.035). Another interesting statistical association is that between right wrist pain and BMI (OR = 1.72, 95% CI = 1.24–2.39, *p* = 0.001); at the same time, hip pain is associated with BMI (OR = 1.47, 95% CI = 1.04–2.07, *p* = 0.030). Gymnastics, unlike many other sports, requires athletes to use their upper extremities to bear large loads, exposing musculoskeletal system to repetitive biomechanical stresses, where it is not usually expected. The lower extremity is also subjected to considerable physical loading, through repetitive impacts on the ground resulting from vault takeoffs and dismounts from different heights, and during tumbling activities [19]. Our evidence is supported by Chawla et al., who noticed that wrist pain and injury are more common among athletes who are older, taller and with a larger BMI [24]. As the age increases, there occurs an increase in difficulty of the skills practiced, as well as an increase in hours and intensity of gymnastics training. This often contributes to the overuse of certain anatomical structures, causing long-term effects. In addition, as the age increases, usually the body weight of young athletes increases too, due to the physiological individual growth, resulting in higher loads on joints, already stressed by overuse [3,27,31,32]. Lastly, older gymnasts are more susceptible to MP or injury than younger ones, because of a prolonged time of exposure to risk [33].

The findings of this research also suggest the need for gymnastics to rethink training programs [34,35], making them more suitable in terms of athletic loads so as to prevent the development of musculoskeletal pain syndromes, in particular those of chronic nature [36,37]. To achieve this, it is desirable to integrate traditional training programs with new technologies, which are increasingly widespread in kinesiology and sports rehabilitation, with therapeutic and preventive purposes [38,39,40,41,42,43,44,45]. These are already used in other areas of rehabilitation, such as neurological rehabilitation [46], in which the new frontiers of telemedicine even provide the possibility of using serious games based on virtual reality for rehabilitative purposes and in order to follow and treat patients remotely [47].

It would be desirable to periodically schedule health screenings and physical examinations for professional gymnasts, both at the beginning of their career and at high levels of competition, with the aim of creating special and tailor-made training programs. These screening measures assume a certain importance in a context where the involvement of athletes at an early age is observed, so it is necessary to identify any individual risk to develop MP or injury as soon as possible [8]. Moreover, physicians and therapists who treat gymnasts’ MP need to be adequately educated about the biomechanical requirements of this sports activity, where overuse pain and injuries involve specific anatomical districts. Benjamin et al. suggest some strategies aimed at the prevention and treatment of wrist pain, according to which physical therapy should include core stabilization exercises, mobility, and stabilization exercises of shoulder and elbow, in order to better redistribute loads during activities involving the upper limbs [2]. In addition, gymnastics coaches should be strongly competent in proper training volume and adequate rest, which are critical to pain and symptom management and recovery. A medical evaluation is advisable at the first manifestation of painful symptoms, which should be considered a warning and therefore promptly investigated for an early detection of developing stress injuries [10,24,27]. Coaches are required to supervise and protect athletes’ practice from early on, and in a gradual progression of technique to more complex executions [27,48,49].

These precautions, combined with greater care of one’s lifestyle, could certainly allow to maintain high levels of sports performance by limiting the risk of developing painful disorders affecting the musculoskeletal system. This way, the general quality of life of gymnasts could also improve, since even the normal activities of daily life would be free from limitations that are due to algo-dysfunctional syndromes. A set of prevention strategies should minimize the risk of MP and injuries due to the competitive nature of the sport, characterized by the ever-increasing physical demands [33,50].

### Limitations

The main limitation of this study is the retrospective and self-reporting nature of the survey questions. The data were therefore provided without any clinical monitoring, and, sometimes, this may have led to an inaccurate definition of the MP by participants. Moreover, the sample is small, even if it refers to a sport whose diffusion is limited compared to other sports.

On the contrary, we consider a strength of this study to be the fact that, to our knowledge, it was the first research to delve into the possible association between MP in all the possible exposed anatomical districts and gymnastics at a professional level.

## 5. Conclusions

Gymnastics is one of the most popular competitive sports in the world, but it also exposes athletes to develop MP and injuries. As observed in this research, many anatomical districts are subjected to MP due to sports overuse, and this is particularly evident for wrists, lumbar spine, and lower limbs.

The more gymnasts practice this sport, especially in terms of long sports practice, the more likely they are to develop pain in many musculoskeletal districts. Increased age and BMI seem to be potential risk factors for arising MP as well. Finally, it is important to understand how, and if, lifestyle habits could affect the MP prevalence among professional gymnasts.

It is desirable that further research delves into gymnastics-related MP and designs new training strategies to prevent it, in order to limit the risk of abandonment and to improve the diffusion of these sports activities, which traditionally allow to develop psychophysical benefits when practiced from a very young age.

## Figures and Tables

**Table 1:** Sample demographic characteristics.

Variable	Value
Females, *n* (%)	78 (98.7%)
Age, mean ± DS (range)	13.7 ± 3.0 (6–21)
Height (mt); mean ± DS (range)	1.55 ± 0.13 (1.20–1.83)
Weight (kg); mean ± DS (range)	44.6 ± 10.0 (24–77)
BMI; mean ± DS (range)	18.4 ± 2.1 (14.1–23.7)
Discipline; *n* (%) Rhythmic gymnastics Artistic gymnastics	54 (68.4) 25 (31.6)
Practice period (months); mean ± DS (range)	80.5 ± 37.0 (0–180)
Number of training sessions per week; mean ± DS (range)	4.1 ± 1.3 (2–8)
Hours spent in a sitting position per day; *n* (%) Less than 2 hours Between 2 and 4 hours Between 4 and 6 hours Between 6 and 8 hours More than 8 hours	19 (24.1) 26 (32.9) 18 (22.8) 15 (19.0) 1 (1.2)

**Table 2:** Prevalence of pain and incidence × 100 months-person, by anatomical district.

District	Prevalence		Incidence × 100 Months-Person		
	*n*	%	95% CI	Inc.	95% CI
Musculoskeletal pain	65	82.9	72.1–90.0	10.2	8.0–13.0
Right hand	0	0.0	0.0–4.6	0.0	-
Left hand	0	0.0	0.0–4.6	0.0	-
Right wrist	15	19.0	11.0–29.4	2.4	1.4–3.9
Left wrist	10	12.7	6.2–22.0	1.6	0.8–2.9
Right elbow	1	1.3	0.3–6.9	0.02	0.01–0.11
Left elbow	0	0.0	0.0–4.6	0.0	-
Right shoulder	9	11.4	5.3–20.5	1.4	0.1–2.7
Left shoulder	9	11.4	5.3–20.5	1.4	0.1–2.7
Cervical spine	0	0.0	0.0–4.6	0.0	-
Dorsal spine	10	12.7	6.2–22.0	1.6	0.8–2.9
Lumbar spine	19	24.1	15.1–35.0	3.0	1.9–4.7
Sacrococcygeal spine	8	10.1	4.5–19.0	1.3	0.1–2.5
Right hip	13	16.5	9.1–26.5	2.0	1.2–3.5
Left hip	9	11.4	5.3–20.5	1.4	0.1–2.7
Right thigh	13	16.5	9.1–26.5	2.0	1.2–3.5
Left thigh	12	15.2	8.1–25.0	1.9	1.1–3.3
Right knee	21	26.6	17.3–37.7	3.3	2.2–5.1
Left knee	21	26.6	17.3–37.7	3.3	2.2–5.1
Right ankle	20	17.7	16.2–36.4	3.1	2.0–4.9
Left ankle	14	17.7	10.0–27.9	2.2	1.3–3.7
Right foot	3	3.8	0.8–10.7	0.05	0.02–0.15
Left foot	2	2.5	0.3–8.8	0.03	0.00–0.13

## Data Availability

The datasets used and/or analysed during the current study will be made available upon reasonable request to the corresponding author, G.F.
